# The Centres for Disease Control light trap (CDC-LT) and the human decoy trap (HDT) compared to the human landing catch (HLC) for measuring *Anopheles* biting in rural Tanzania

**DOI:** 10.1186/s12936-022-04192-9

**Published:** 2022-06-11

**Authors:** Isaac Haggai Namango, Carly Marshall, Adam Saddler, Amanda Ross, David Kaftan, Frank Tenywa, Noely Makungwa, Olukayode G. Odufuwa, Godfrey Ligema, Hassan Ngonyani, Isaya Matanila, Jameel Bharmal, Jason Moore, Sarah J. Moore, Manuel W. Hetzel

**Affiliations:** 1 Swiss Tropical and Public Health Institute, Allschwil, Switzerland; 2 University of Basel, Basel, Switzerland; 3 Vector Control Product Testing Unit, Ifakara Health Institute, Bagamoyo, Tanzania; 4 British Columbia Centre for Excellence in HIV/AIDS, Vancouver, British Columbia Canada; 5 Telethon Kids Institute, Perth, Australia; 6 New York University Grossman School of Medicine, New York, NY USA; 7 London School of Hygiene and Tropical Medicine, London, UK; 8 Innovative Vector Control Consortium, Dar es Salaam, Tanzania; 9 Nelson Mandela African Institute of Science and Technology, Arusha, Tanzania

**Keywords:** Mosquito traps, *Anopheles* biting, Entomological monitoring

## Abstract

**Background:**

Vector mosquito biting intensity is an important measure to understand malaria transmission. Human landing catch (HLC) is an effective but labour-intensive, expensive, and potentially hazardous entomological surveillance tool. The Centres for Disease Control light trap (CDC-LT) and the human decoy trap (HDT) are exposure-free alternatives. This study compared the CDC-LT and HDT against HLC for measuring *Anopheles* biting in rural Tanzania and assessed their suitability as HLC proxies.

**Methods:**

Indoor mosquito surveys using HLC and CDC-LT and outdoor surveys using HLC and HDT were conducted in 2017 and in 2019 in Ulanga, Tanzania in 19 villages, with one trap/house/night. Species composition, sporozoite rates and density/trap/night were compared. Aggregating the data by village and month, the Bland–Altman approach was used to assess agreement between trap types.

**Results:**

Overall, 66,807 *Anopheles funestus* and 14,606 *Anopheles arabiensis* adult females were caught with 6,013 CDC-LT, 339 indoor-HLC, 136 HDT and 195 outdoor-HLC collections. Indoors, CDC-LT caught fewer *An. arabiensis* (Adjusted rate ratio [Adj.RR] = 0.35, 95% confidence interval [CI]: 0.27–0.46, p < 0.001) and *An. funestus* (Adj.RR = 0.63, 95%CI: 0.51–0.79, p < 0.001) than HLC per trap/night**.** Outdoors, HDT caught fewer *An. arabiensis* (Adj.RR = 0.04, 95%CI: 0.01–0.14, p < 0.001) and *An. funestus* (Adj.RR = 0.10, 95%CI: 0.07–0.15, p < 0.001) than HLC. The bias and variability in number of mosquitoes caught by the different traps were dependent on mosquito densities. The relative efficacies of both CDC-LT and HDT in comparison to HLC declined with increased mosquito abundance. The variability in the ratios was substantial for low HLC counts and decreased as mosquito abundance increased. The numbers of sporozoite positive mosquitoes were low for all traps.

**Conclusions:**

CDC-LT can be suitable for comparing mosquito populations between study arms or over time if accuracy in the absolute biting rate, compared to HLC, is not required. CDC-LT is useful for estimating sporozoite rates because large numbers of traps can be deployed to collect adequate mosquito samples. The present design of the HDT is not amenable for use in large-scale entomological surveys. Use of HLC remains important for estimating human exposure to mosquitoes as part of estimating the entomological inoculation rate (EIR).

**Supplementary Information:**

The online version contains supplementary material available at 10.1186/s12936-022-04192-9.

## Background

Measuring *Anopheles* biting is a core part of the monitoring and surveillance of malaria vectors. The *Anopheles* females, responsible for the transmission of malaria, incidentally ingest or inoculate malaria parasites while biting humans to obtain a blood meal needed for egg production [1, 2]. The proportion of biting mosquitoes that are infected is essential to quantify the entomological inoculation rate (EIR). The EIR, the number of infectious bites per person per time unit, is a standard vector-based index for estimating transmission intensity [3, 4]. *Anopheles* biting is assessed by collecting host-seeking mosquitoes around areas occupied by humans over regular time intervals throughout the night [5].

**Fig. 1:**
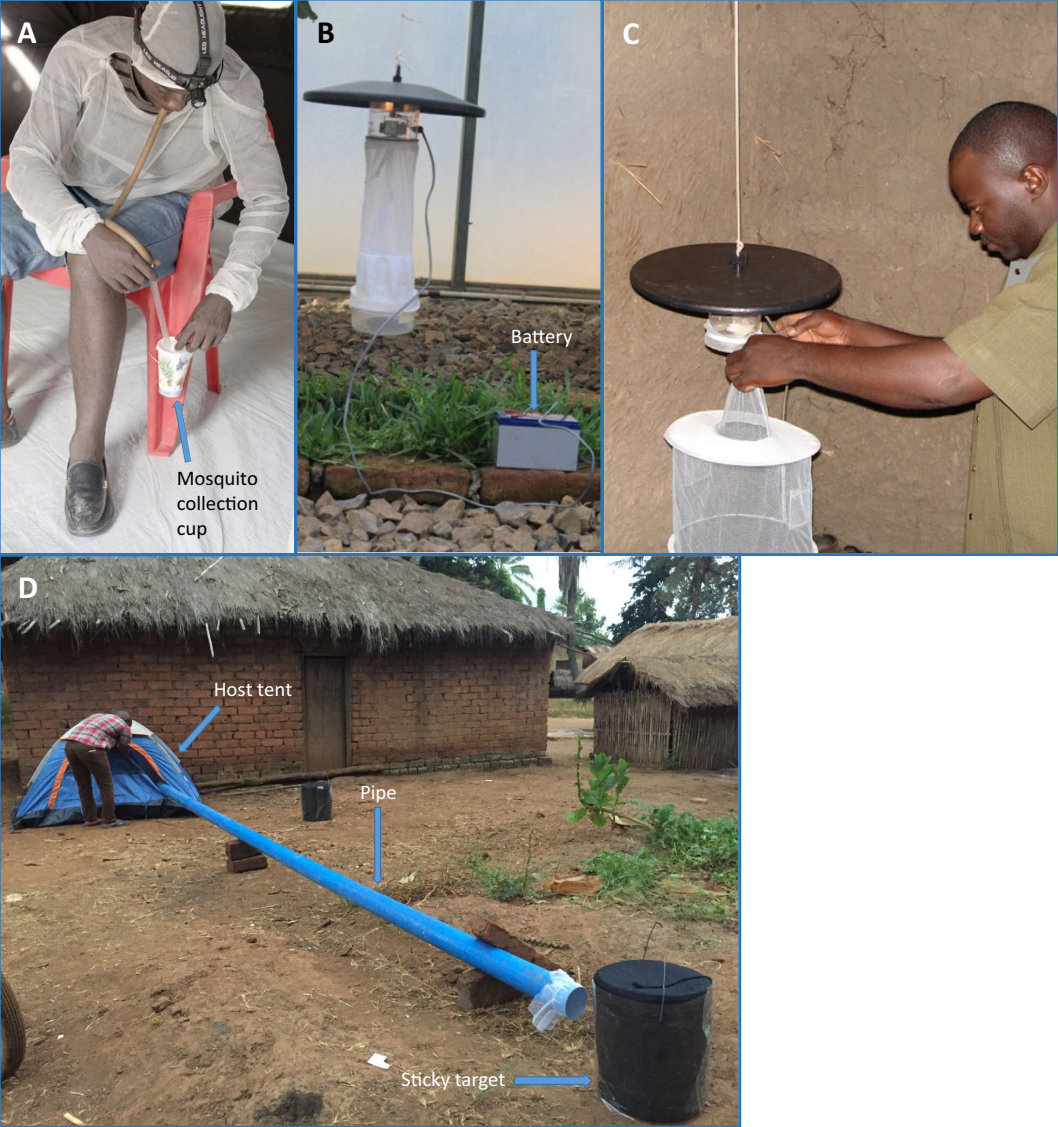
Illustrations of mosquito traps. **A** The human landing catch (HLC) technique showing a catcher transferring a trapped mosquito that they have aspirated from their lower limb into a collection container. **B** The standard CDC-LT (Model 512; John W. Hock Company, Gainesville, FL). **C** A study field assistant setting up a CDC-LT inside a house at the foot of an occupied bed net with the entry point of the trap 70 cm from the ground. **D** The human decoy trap (HDT). A study field assistant preparing the tent to be occupied by a human. Host odour emanating from a protected human in the tent positioned nearby is blown by a battery-powered computer fan down the connecting pipe and delivered around the sticky black target. The sticky target consists of a cylindrical container, dark in colour to improve visual contrast, and is wrapped by an adhesive transparent plastic material. The target is augmented by filling it with warm water kept at 35 ± 5 °C by a heating mechanism, to give mosquitoes a combination of olfactory cues from the host odour as well as heat. Host-seeking mosquitoes lured by heat and smell and the dark colour around the target are trapped on its sticky surface

The human landing catch (HLC), is considered the gold-standard method to assess human exposure to *Anopheles* biting [6, 7]. Individuals recruited to perform HLC (catchers), collect mosquitoes attracted to and alighting on their lower limbs with the help of an aspiration tube before they are able to bite (Fig. 1). The catchers collect mosquitoes ideally over hourly intervals all night [5, 8]. The number of mosquitoes collected by the HLC is presumed to represent the actual intensities and hourly patterns of malaria vectors biting humans. The human biting rate and the EIR assessed by the HLC have for a long time been considered to be the most appropriate for malaria surveillance and are used as a reference for standardizing other methods [7, 9].

HLC surveys, however, have important limitations that restrict use. Although it has been demonstrated that observing proper HLC protocol minimizes the risk of malaria infection among catchers [10, 11], use of human baits to catch mosquitoes that have the potential to transmit disease has ethical concerns [12]. The safety of catchers is compromised with the emergence of anti-malarial drug-resistance or in areas with active arbovirus circulation [13]. Surveys by the HLC are also labour-intensive, cumbersome and incur considerable costs to run on a large scale [14]. In addition, variations in the alertness and skill of catchers requires careful supervision, and differences in human attractiveness to mosquitoes make HLC surveys hard to standardize [15] As such, the HLC has been found unsuitable for extensive use in monitoring malaria vectors for disease control [16]. The World Health Organization (WHO) encourages research and use of alternative mosquito traps with only sparing use of HLC for purposes such as calibrating new tools [17, 18].

Several attempts have been made to find options that measure biting rates but do not rely heavily on human effort or risk exposure to infection [7, 15, 19]. The target profile for anopheline collection methods to be used as HLC surrogates comprise traps that actively lure host-seeking females by use of host-based cues. Most trap technologies utilize a combination of olfactory, visual and thermal cues and the suitability of a trap relies on the effectiveness to replicate human attraction [20]. The number of mosquitoes caught by a trap should either be the same as or comparable via an equation to those caught by the HLC [20]. Ideally, the traps would collect similar mosquito samples as the HLC, including same species composition, age range and the proportion of infected mosquitoes [6]. Entomological monitoring for malaria transmission control further requires traps that are low cost and easily scalable.

Developed initially for sampling agricultural pests, light traps have found common use as anopheline traps after Odetoyinbo first demonstrated their efficacy [21]. The most common light trap is the battery-powered Centres for Disease Control light trap (CDC-LT) shown in Fig. 1. This trap is often used alongside occupied sleeping spaces protected by untreated bed nets and exploit odour cues emanating from the sleeping individuals to attract mosquitoes [22–24]. Although the exact trapping mechanism of the CDC-LT-bed net combination is not well known, it is thought that mosquitoes which persistently attempt to enter the bed nets are eventually caught as they fly close to the trap [25]. The trap performance is greatly enhanced if they are placed in the normal flight paths of mosquitoes close to the feet of sleeping people [25] as well as inside huts or under eaves [21]. Compared to the HLC, CDC-LTs are easy to use, have considerably lower costs to operate, are easily scalable and standardized. Mechanical malfunctioning and battery problems, highlighted as the main limitations of these traps usually occur on a minimal scale and faulty traps are often conveniently excluded from mosquito surveys [22]. CDC-LTs are also used for outdoor mosquito catches, but they tend to perform poorly outdoors compared to indoors [26]. Although generally regarded as a reliable mosquito trap [22, 27] there is no clear consensus on the CDC-LT performance relative to the HLC. The CDC-LT performance appears to vary based on the local settings [28–30].

The host or human decoy trap (HDT) was first trialled against *Anopheles* mosquitoes in an attempt to cover a malaria vector monitoring gap for outdoor biting populations in Burkina Faso [31]. The HDT optimizes mosquito attraction by use of a combination of odour and visual stimuli and a thermal signature in the range equivalent to the human body temperature. The trap consists of three key parts; a tent, a plastic connecting pipe and a sticky target (Fig. 1). Host odour emanating from a protected human in the tent positioned nearby is blown by a battery-powered computer fan down the connecting pipe and delivered around the sticky black target. The sticky target consists of a cylindrical container, dark in colour to improve visual contrast, and is wrapped by an adhesive transparent plastic material. The target is augmented by filling it with warm water kept at 35 ± 5 °C by a heating mechanism, to give mosquitoes a combination of olfactory cues from the host odour as well as heat. Host-seeking mosquitoes lured by heat and smell and the dark colour around the target are trapped on its sticky surface. The HDT is a promising entomological surveillance tool based on several studies that demonstrate its capacity to catch a wide range of exophagic mosquito species [32–36].

The present study focused on human biting rates of malaria vectors. The CDC-LT and the HDT were compared to the HLC with an overall aim of determining if the traps could replace the HLC for measuring human biting rates in Ulanga, Tanzania. The current study was interested in whether this calibration was accurate at the population level rather than the individual level as has been done in many other studies (Table 1).

**Table 1:** Summary of past studies of the efficacy relative to HLC of the CDC-LT and HDT against *Anopheles* species

No.	Area of study	Dominant anophelines	Relative efficacy: Ratio to HLC (95% confidence intervals)				Was trap efficacy dependent on mosquito density?	References
A. CDC-LT								
i. Mosquito species								
1	Ulanga, Tanzania	*An. arabiensis* *An. funestus*	0.35 (0.27–0.46) 0.63 (0.51–0.79)				Yes Yes	This study
2	Ulanga, Tanzania	*98% An. gambiae s.l* *2% An. funestus*	0.33 (0.24–0.46) 0.82 (0.61–1.10)				Not assessed	Okumu et al. 2008 [59]
3	Kenya, Zambia, Burkina Faso, Ghana, Tanzania	*An. gambiae s.l* *An. funestus*	1.06 (0.68–1.64) 1.37 (0.70–2.68)				Yes Yes	Briët et al*.* 2015 [15]
4	Lwanda, Kenya	*74% An. gambiae s.l* *26% An. funestus*	1.86 (1.73–2.00) 1.91 (1.66–2.19)				No No	Mathenge et al. 2004 [60]
5	Ahero, Kenya	*An. arabiensis* *An. funestus*	0.56 (0.49–0.66) 1.19 (1.03–1.37)				Yes Yes	Mathenge et al. 2005 [30]
6	Rarieda, Kenya	*An. gambiae s.l* *An. funestus*	1.18 (0.55–2.54) 0.69 (0.49–0.98)				Not assessed	Wong et al. 2013 [20]
ii. ITNs vs. no ITNs								
			With ITNs		Without ITNs			
7	Bo, Sierra Leone	*An. gambiae s.l*	0.88 (0.72–1.05)		0.78 (0.60–1.01)		No (without ITNs) Yes (with ITNs)	Magbity et al. 2002 [27]*†
iii. Indoors vs. outdoors								
			Indoors		Outdoors			
8	Wosera, Papua New Guinea	*An. koliensis* *An. panctulatus* *An. karwari* *An. farauti* s.l *An. longirostris* *An. bancroftii*	0.28 (0.27–0.29) 0.10 (0.09–0.11) 0.12 (0.11–0.13) 0.07 (0.06–0.09) 0.12 (0.08–0.15) 0.20 (0.15–0.27)		0.27 (0.26–0.28) 0.09 (0.08–0.09) 0.12 (0.11–0.13) 0.06 (0.05–0.08) 0.07 (0.05–1.05) 0.15 (0.11–0.20)		Yes Yes Yes Yes Yes Yes	Hii et al. 2000 [58]
9	Bioko Island, Equatorial Guinea	*An. gambiae s.s* & *An. melas*	0.12 (0.11–0.14) (Mongola area) 0.36 (0.32–0.40) (Arena Blanca area) 0.13 (0.10–0.16) (Riaba area)		0.009 (0.01–0.012) (Mongola area) 0.10 (0.09–0.12) (Arena Blanca area) 0.07 (0.05–0.09) (Riaba area)		Yes (indoors) No (outdoors) Yes Yes	Overgaard et al. 2012 [55]*
iv. Location								
			Kakola-Ombaka area		Masogo area			
10	Nyando & Muhoroni, Kenya	*An. arabiensis* *An. funestus* *An. coustani*	1.98 (1.01–3.86) 0.88 (0.37–2.11) 3.03 (1.65–5.56)		1.83 (0.70–4.79) 0.45 (0.13–1.57) 2.88 (1.15–7.22)		Not assessed	Abong’o et al. 2021 [32]
B. HDT								
1	Ulanga, Tanzania	*An. arabiensis* *An. funestus*	0.04 (0.01–0.14) 0.10 (0.07–0.15)				Yes Yes	This study
i. Type of host bait								
			Cow-baited		Human-baited			
2	Kisumu & Homa Bay, Kenya	*An. gambiae* s*.s* & *An. arabiesnsis* & *An. funestus* & *An. coustani*	7.08 (Kisian) 8.34 (Homa Bay)		0.17 (Kisian) 0.60 (Homa Bay)		Not assessed	Abong’o et al. 2018 [35]*
ii. Location								
			Kakola-Ombaka area		Masogo area			
3	Nyando & Muhoroni, Kenya	*An. arabiensis* *An. funestus* *An. coustani* *An. pharoensis*	5.69 (2.98–10.86) 1.38 (0.60–3.18) 0 18(0.09–0.37) NA		1.32 (0.49–3.59) 0.66 (0.21–2.09) 2.88 (1.15–7.22) NA		Not assessed	Abong’o et al. 2021 [32]
			Lakkang area		Pucak area			
4	Chikwawa, Malawi	*An. gambiae s.s* & *An. Arabiensis* & *An. coustani* & *An. quadriannulatus* & *An. tenebrosus*	1.03 (0.80–1.30)		0.83–3.17)		Not assessed	Zembere et al. 2021 [33]*
iii. Season								
			Rainy season	Early dry season		Late dry season		
5	Vallée de Kou, Burkina Faso	*An. gambiae* *An. pharoensis* *An. coustani*	9.6 (9.4–9.7) 10.5 (10.4–10.7) NA	2.2 (2.0–2.4) 2.8 (2.5–3.0) 18.6 (18.2–19.1)		1.7 (1.3–2.0) 1.7 (1.3–2.1) NA	Not assessed	Hawkes et al. 2017 [31]

*NA* not assessed because of data scarcity

*Ratio estimated for pooled mosquito species

^†^Three CDC-LTs were compared to two HLC catchers

## Methods

### Study area

**Fig. 2:**
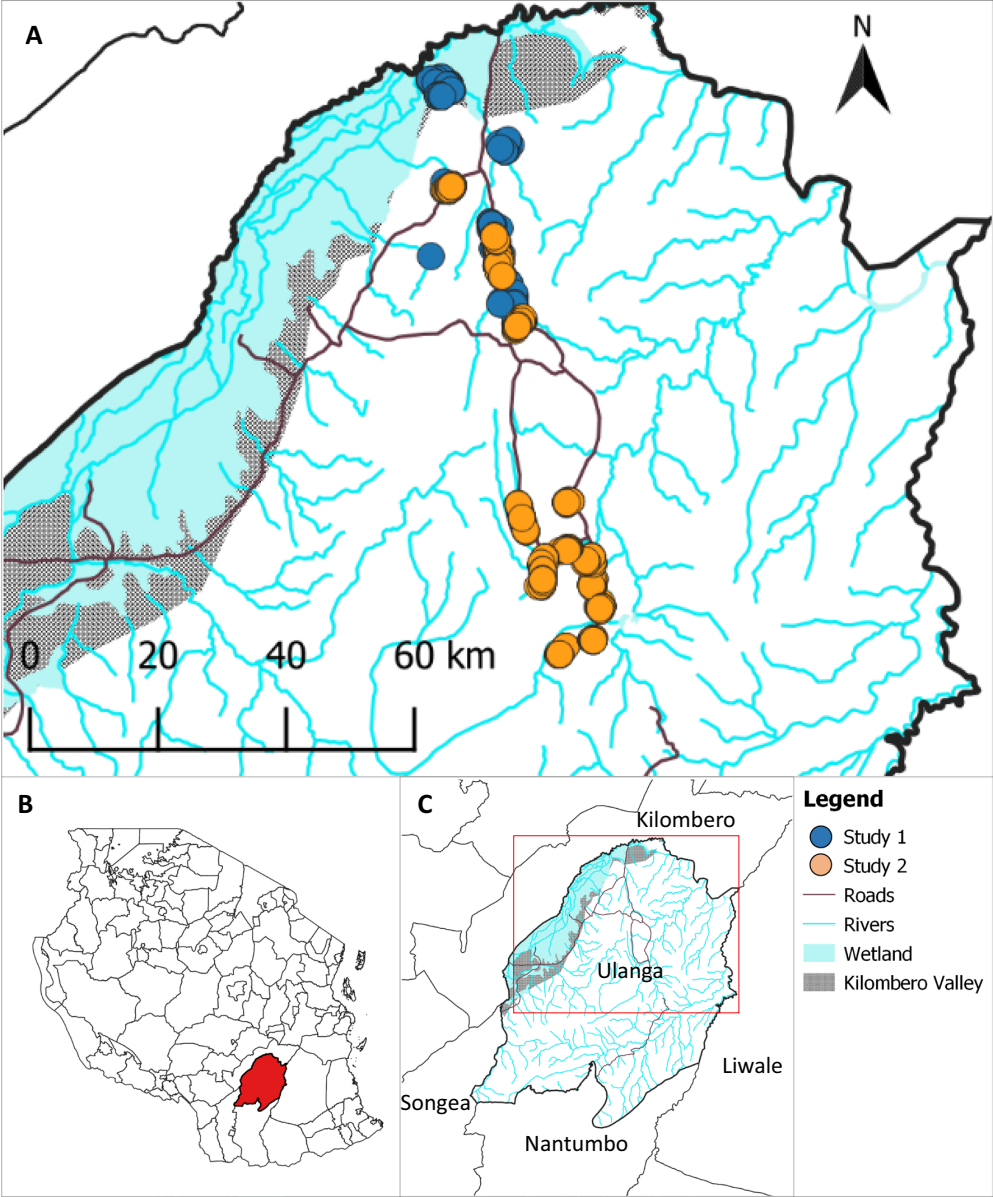
Map of the study area. **A** shows house locations where mosquito surveys were conducted. Overlapping dots represent closely located households. **B**, **C** show the locations of Ulanga District in Tanzania and of the study area in Ulanga District, respectively

The study area was in Ulanga District, south-eastern Tanzania (Fig. 2). Ulanga is located in the wider Kilombero River floodplain. The region is characterized by a hot-humid climate, seasonal floodplains and irrigated rice paddies. The main malaria vectors are *Anopheles funestus* and *Anopheles arabiensis* [37–40]. Moderate levels of malaria transmission occur all year with peak in transmission intensities experienced around the rainy season (January to May) [41, 42]. Increased use of ITNs as the primary malaria protection measure in the communities is thought to have contributed to significant reductions in malaria transmission [42–44].

### Study design

Two community randomized studies to evaluate the effectiveness of two new indoor residual spraying (IRS) products were conducted in 2017 (Study 1) and in 2019 (Study 2). Detailed descriptions of the IRS trials are presented in two papers (in preparation). The mosquito surveys were performed by HLC, the CDC-LT and the HDT. Population clusters (villages) were selected close to rice paddies where high mosquito densities were presumed to occur, and the study houses were randomly selected within the villages using a random number generator. The villages were all similar in their altitudes, house types, use of vector control tools and socio-economic status of households, although some villages may have had more houses than others. Overall, 19 villages were surveyed; Study 1 covered ten villages while Study 2 covered 14 villages that partly overlapped five villages from Study 1. The villages were paired into intervention and control arms and were separated by at least 2 km to limit mosquito migration between treatment arms. House surveys were conducted to collect data on household characteristics such as the number of occupants, number of sleeping spaces, presence of livestock, materials used on house walls, roof, ceiling, floor, and condition of eaves, window and door screening. The global positioning system (GPS) coordinates for house locations were recorded for all surveyed households. Sampling for different traps was based on monthly mosquito population estimates at the cluster level and not designed to occur simultaneously for the same nights or for the same houses. Traps were randomly assigned to houses in the villages. However, on some occasions, sampling for different traps was done on the same nights but in different houses. The study periods involving all traps covered the rainy (January to May) and the dry (July to December) seasons.

### Human landing catch (HLC) collections

The HLC surveys followed WHO guidelines [45]. Two catchers collected mosquitoes indoors and outdoors, alternating positions every hour. Collections were performed for 45 min followed by 15 min break. The catchers received doxycycline for malaria prophylaxis and were tested weekly for malaria infection using Bioline Malaria Ag Pf/Pan rapid diagnostic tests. Mosquitoes were collected from 18:00PM to 06:00AM in three randomly selected houses per village per night. The surveys were repeated for six nights per month for five months in Study 1 and for eight months in Study 2.

### CDC-LT collections

The standard miniature CDC-LT (Model 512; John W. Hock Company, Gainesville, FL.) was used for the surveys (Fig. 1). Traps were set indoors at sleeping spaces protected by untreated bed nets, at the foot end of the bed, with the light source positioned at approximately 70 cm from the ground as described by Mboera et al. [25]. The traps were operated from 18:00 PM to 06:00 AM in three randomly selected houses per village in Study 1 and in four randomly selected houses per village in Study 2. The traps were used for six nights per month for five months in Study 1 and for 20 nights per month for 8 months in Study 2.

### Human decoy trap (HDT) collections

The HDT used in this study was a modification of the standard Biogents, Regensburg, Germany, developed as described by Hawkes et al. [46] and is shown in Fig. 1. HDT surveys were conducted as described by Hawkes et al. [31] and in accordance with the WHO general guidelines [45]. The traps were operated outdoors between 18:00PM to 06:00AM in 4 randomly selected houses per village and were repeated monthly for up to 3 months. The HDT surveys were only done in Study 2.

### Sorting and molecular identification of mosquitoes

Female adult anophelines were morphologically identified and separated at genus and species complex level by field technicians. Polymerase chain reaction (PCR) was used to identify sibling species within the *An. funestus* [47] and the *Anopheles gambiae* [48] complexes.

### Sporozoite detection in mosquito salivary glands

Enzyme linked immunosorbent assays (ELISA) was used for detection of *Plasmodium falciparum* circumsporozoite protein (Pf CSP) in the salivary glands of mosquitoes [49]. Detection of *P. falciparum* parasites was performed from heads and thoraxes for pooled mosquito samples, separately for *An. arabiensis* and *An. funestus*. Sample pooling was done by house ID, date and hour of collection and by trap type and did not exceed twenty mosquitoes. The number of mosquitoes per pool was recorded. The optical density of post-ELISA lysate were measured at 405–414 nm after 45 min using ELISA plate reader machine [50].

### Data analysis

Violin plots were used to display the distribution of the number of mosquitoes caught per trap per night. Due to skewness, the counts were log transformed by first adding a value of 1 to the number of mosquitoes (density) per trap per night i.e. log (density + 1). Nightly trap catches were summarized using geometric means with 95% confidence intervals (95% CIs) and medians with 90% central ranges. The relative proportions of *An. funestus and An. arabiensis* mosquito species caught by the traps were estimated using a logistic regression models with a random effect for house and date. The association between trap type and the number of mosquitoes caught was estimated by the negative binomial-generalized linear mixed-effects models (negative binomial-GLMMs); with random effects for house and date and fixed effects for household size, livestock and pets, house screening, IRS treatment, ITNs use, seasonality, and whether the measurements were taken as part of Study 1 or 2 (only for CDC-LT versus indoor HLC). A series of similar models were fitted regressing mosquito counts on species type to determine the relative abundance of species per trap per night. Since the HDT surveys were only done in Study 2, the trap was compared to outdoor HLC surveys restricted to Study 2 (Additional file 1: Tables S1, S2 and S3).

Agreement for individual catches could not be assessed since there were no paired observations for the same households and nights. Instead, collections were aggregated by village and month (village-month) to calculate the geometric mean number of mosquitoes caught per house per night for each trap.

The Bland and Altman approach [51] was used to assess agreement between the trap types, providing estimates of the overall bias and the variability. The bias was measured by the ratio of the geometric mean for each trap type (HDT or CDC-LT) compared to the geometric mean using HLC, calculated for the village-months. The ratios were logarithmically transformed (because the distribution of the ratios was skewed). The log ratios were then plotted against the HLC density [52, 53]. The HLC density rather than the mean of two trap types were used because HLC was considered to be a gold standard [52]. The estimates of the variability were presented as 95% limits of agreement, which represent the range in which 95% of the ratios are expected to lie.

To investigate the effect of density on agreement, the regression approach as described by Bland and Altman [54] was used. To investigate the effect of the trap on the mean ratio by density, a regression model was fitted with the log ratio as the outcome variable and HLC density as the explanatory variable. This way, an estimate of the effect of mosquito densities on the ratio of the geometric means of CDC-LT (or HDT) to HLC could be obtained. The effect of mosquito densities on the variability and limits of agreement was estimated by regressing the absolute values of the residuals of the previous model on HLC catches. Village-months with ten or less CDC-LT and indoor HLC collection pairs were excluded from the agreement analysis due to stochasticity.

The prevalence of Pf CSP ELISA positive mosquitoes was estimated for each trap type. Due to a very low sporozoite prevalence, no comparative statistical analyses were made between the traps.

The statistical analyses were performed in Stata (16.1, StataCorp LLC, College Station, TX) and in R version 4.0.3 (R Foundation for Statistical Computing, Vienna, Austria).

## Results

**Fig. 3:**
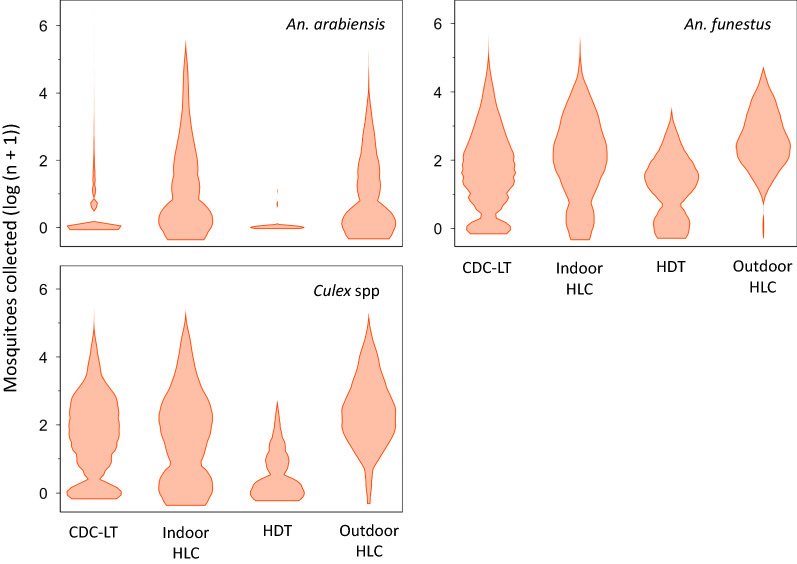
Density distribution of log nightly mosquito catches per trap. The violin plots were plotted from log transformed mosquito numbers due to skewness. Because of zeros in the data, a value of 1 was added to the nightly numbers of mosquitoes prior to the logarithmic transformation

Altogether, there were 6,013 CDC-LT, 339 indoor HLC, 136 HDT and 195 outdoor HLC collections. A greater number of *An. funestus* (66,807) than *An. arabiensis* (14,606) adult females were caught. The traps also caught a total 75,248 *Culex* spp mosquitoes, mostly a common source of biting nuisance throughout the tropics. The number of mosquitoes collected per trap per night were generally low across all traps throughout the study, with a skewed distribution (Fig. 3). The skew was largely due to collections when no mosquitoes were caught by either of the traps but were included in the analysis since such observations are frequently encountered in natural populations. There was substantial variation in the number of mosquitoes caught per trap per night. Table 2 shows details of the surveys and compares mosquito catches between traps.

**Table 2:** The estimated total and nightly mean catch by CDC-LT and the human decoy trap (HDT) compared to the human landing catch (HLC)

		*An. arabiensis*						*An. funestus*						*Culex* spp					
Trap type	Total collections	Total caught	Geometric mean (95%CI)	Median (90% central range)	Range	Adj.RR† (95%CI)	p-value	Total caught	Geometric mean (95%CI)	Median (90% central range)	Range	Adj.RR† (95%CI)	p-value	Total caught	Geometric mean (95%CI)	Median (90% central range)	Range	Adj.RR† (95%CI)	p-value
Indoor HLC	339	3380	2.39 (1.93–2.91)	1(0–61)	0–180	1		3934	4.22 (3.53–5.02)	5(0–58)	0–147	1		4803	6.51 (5.58–7.57)	7(0–46)	0–196	1	
CDC-LT	6013	10,281	0.51 (0.48–0.54)	0(0–7)	0–658	0.35 (0.27–0.46)	< 0.001	59,276	4.61 (4.45–4.78)	5(0–39)	0–240	0.63 (0.51–0.79)	< 0.001	66,459	5.01 (4.84–5.19)	5(0–45)	0–250	0.10 (0.07–0.15)	< 0.001
Outdoor HLC	195	940	1.56 (1.18–2.00)	1(0–23)	0–139	1		3408	4.14 (3.48–4.91)	9(1–63)	0–143	1		3460	11.71 (10.15–13.48)	11(2–58)	0–86	1	
HDT	136	5	0.02 (0.00–0.05)	0(0–0)	0–2	0.04 (0.01–0.14)	< 0.001	189	0.77 (0.56–0.99)	0(0–7)	0–11	0.10 (0.07–0.15)	< 0.001	526	2.33 (1.86–2.88)	3(0–12)	0–25	0.20 (0.14–0.29)	< 0.001

The geometric means were computed by exponentiating the arithmetic means of the log transformed nightly catches per trap. A value of 1 was added to the nightly figures of caught mosquitoes prior to the logarithmic transformation i.e. log (density + 1)

†The adjusted mosquito sampling rate ratios (Adj. RR) and 95% confidence intervals (95%CI) were estimated from negative binomial regression models. The models included random effects for day and house, and fixed effects for the household size, livestock, house screening, IRS treatment, ITNs use, season (rainy or dry), and Study (1 or 2)

1* reference method

Aggregating the trap collections per village-month gave a total of 116 CDC-LT and indoor HLC pairs with a median of 66 (90% central range (CR): 6–89) collections per village-month and 40 HDT and outdoor HLC pairs with a median of 6 (90% CR: 4–9).

**Fig. 4:**
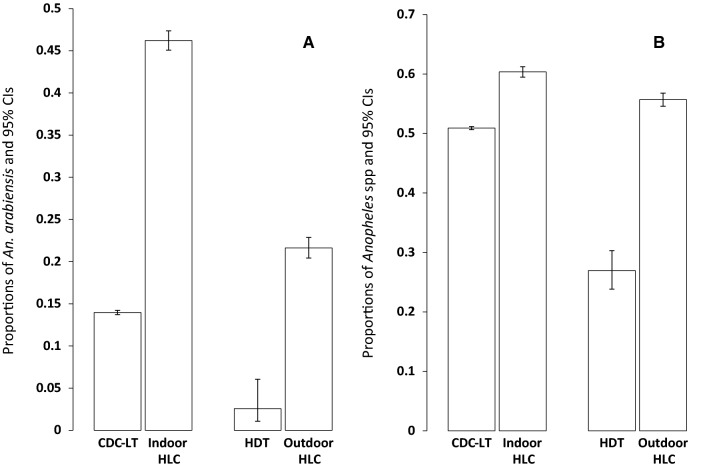
The proportions of *Anopheles* mosquitoes caught by traps. The relative proportions of (**A**) *An. arabiensis* versus *An. funestus* and (**B**) *Anopheles* spp versus *Culex* spp were estimated from logistic regression models adjusted for random effects of house and date. (The error bars represent 95% confidence intervals (CI)). The relative proportion of *An. arabiensis* compared to *An. funestus* was lower for CDC-LT collection than HLC (odds ratio [OR] = 0.24, 95% CI = 0.20–0.29, p < 0.001). The proportion of *Anopheles* spp compared to *Culex* was lower for CDC-LT collection than HLC (OR = 0.90, 95% CI = 0.77–1.04, p < 0.001). The relative proportions of *An. arabiensis* and *Anopheles* spp were also lower estimated by the HDT (OR = 0.11, 95% CI = 0.04–0.28, p < 0.001) and (OR = 0.34, 95% CI = 0.26–0.47, p < 0.001) respectively, compared to those of the outdoor HLC

The relative proportions of *An. arabiensis* compared to *An. funestus* and *Anopheles* spp compared to *Culex* spp were lower for CDC-LT collection than HLC (odds ratio [OR] = 0.24, 95% confidence interval [CI]: 0.20–0.29 p < 0.001 and OR = 0.90, 95% CI: = 0.77–1.04, p < 0.001, respectively). The relative proportions of *An. arabiensis* and *Anopheles* spp estimated by the HDT were also lower than those of the outdoor HLC (OR = 0.11, 95% CI = 0.04–0.28, p < 0.001 and OR = 0.34, 95% CI: = 0.26–0.47, p < 0.001, respectively) (Fig. 4).

The CDC-LTs caught approximately a third as many *An. arabiensis* (Adjusted rate ratio (Adj.RR) = 0.35, (95%CI: 0.27–0.46, p < 0.001) and about two-thirds as many *An. funestus* (Adj.RR = 0.63, 95% CI = 0.51–0.79, p < 0.001) compared to indoor HLC per trap per night**.** The HDT caught far lower numbers of *An. arabiensis* (Adj.RR = 0.04, 95% CI: 0.01–0.14, p < 0.001) and *An. funestus* (Adj.RR = 0.10, 95% CI: 0.07–0.15, p < 0.001) compared to the outdoor HLC. The estimated rate ratios for CDC-LT and HDT for *Culex* spp were 0.82, 95% CI: 0.67–1.01 and 0.20, 95% CI: 0.14–0.29, respectively (Table 2).

All traps caught more *An. funestus* than *An. arabiensis* per night (Additional file 1: Table S4).

**Fig. 5:**
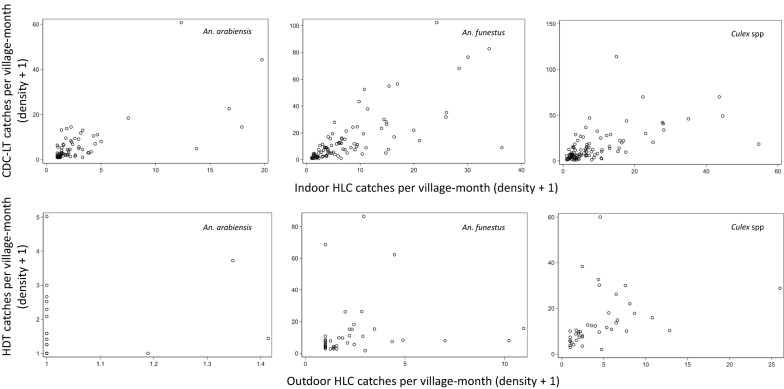
Geometric mean mosquito catches per village-month by the CDC-LT and indoor HLC (upper panels) and by HDT and outdoor HLC (lower panels)

**Fig. 6:**
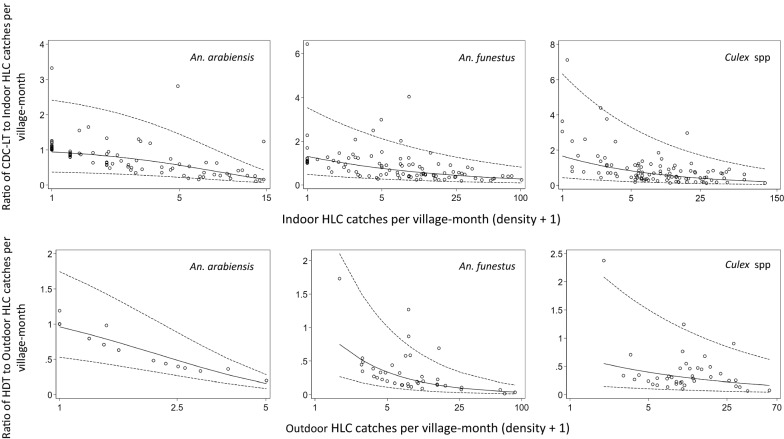
Bland–Altman-based plots showing agreement between CDC-LT and indoor HLC (upper panels) and between HDT and outdoor HLC (lower panels). The solid lines (—) represent the mean ratios of geometric mean catches for the village-month for CDC-LT or HDT compared to HLC (the overall bias). The ratios of geometric catches per village-month were obtained by exponentiating the differences of the logarithms of arithmetic means per village-month between CDC-LT or HDT and HLC i.e. (exp (log (CDC-LT + 1)—log (Indoor HLC + 1)) for CDC-LT to indoor HLC or exp (log (HDT + 1)—log (Outdoor HLC + 1)) for HDT to outdoor HLC). The regression equations used to estimate the overall biases are the translation algorithms that account for the density-dependence of the CDC-LT or HDT effects relative to the HLC. The dotted lines (----) represent the 95% limits of agreement, in which 95% of the ratios were expected to lie

Geometric mean mosquito catches per village-month tended to be higher by the CDC-LT when there were higher catches by indoor HLC and similarly between the HDT and the outdoor HLC (Fig. 5), although there was substantial variation. The mean ratios of geometric means of HDT or CDC-LT to HLC, and the limits of agreement of the ratios, were dependent on mosquito density for all species (Fig. 6). The mean ratios decreased significantly with higher HLC catches, suggesting that the HDT and CDC-LT catch relatively fewer mosquitoes compared to the gold standard HLC at higher mosquito densities. This suggests a density dependent bias that could either be due to mechanical limitations of the traps or greater vigilance among those conducting HLC when mosquito densities are greater. The limits of agreement for the village-months were wide across most of the range of HLC densities in this study but decreased for higher densities.

*Plasmodium falciparum* sporozoite infection rates (SRs) for *An. arabiensis* and *An. funestus* caught by the different traps were low (Table 3). Only CDC-LTs caught any infected *An. arabiensis* mosquitoes and estimated a higher prevalence of infected *An. funestus* compared to indoor HLC. HDT did not catch any infected mosquitoes.

**Table 3:** *Plasmodium falciparum* sporozoite infection rates (SRs) for *An. arabiensis* and *An. funestus* collected by different traps

	Malaria vectors positive by ELISA test								
	*An. arabiensis*			*An. funestus*			Total		
Trap type	Positive	Tested	% Positive	Positive	Tested	% Positive	Positive	Tested	% Positive
Indoor HLC	0	286	0	12	998	1.20	12	1284	0.93
CDC-LT	10	1461	0.68	255	5701	4.47	265	7162	3.70
Outdoor HLC	0	335	0	10	966	1.04	10	1301	0.77
HDT	0	3	0	0	39	0	0	42	0

% positive represents the number of mosquitoes with a positive *P. falciparum* circumsporozoite protein (CSP) ELISA test divided by the total number of mosquitoes tested

*ELISA* Enzyme-linked immunosorbent assay

## Discussion

Monitoring malaria vectors requires accurate, safe and reliable collection methods that can be deployed at scale. It is particularly difficult to collect representative samples of field populations and nearly all sampling tools will have some bias [6, 9]. The choice of a suitable tool depends on the entomological parameters being investigated and the behaviours of local vector populations. The overall target being to select the most appropriate methods that deliver the most meaningful and pertinent information.

The present study focused on human biting rates of malaria vectors. The CDC-LT and the HDT were compared to the HLC with an overall aim of determining if the traps could replace the HLC for measuring human biting at the population level in Ulanga, Tanzania. The HLC collects mosquitoes in the very act in which the medical entomologist is most interested, namely the act of biting a person [22]. It therefore provides the number of mosquitoes that would normally be attracted to an average human host in a night [6, 7, 9]. The CDC-LT and the HDT do not involve direct mosquito-human contact. Therefore, interpreting their catches in terms of human exposure needs to be calibrated against the HLC [9]. The current study was interested in whether this calibration was accurate at the population level rather than the individual level as has been done in many other studies (Table 1).

Controlling for other effects influencing mosquito densities, the CDC-LT caught roughly a third as many *An. arabiensis* and about two thirds as many *An. funestus* as the indoor HLC per trap per night, while the HDT barely caught a tenth of any of these species compared to the outdoor HLC. Using the Bland–Altman method for assessing agreement in matched village-month collections, the CDC-LT and the HDT underestimated *An. arabiensis* and *An. funestus* biting and their performances were poorer at high mosquito densities. One possible explanation for this trend would be reduced attentiveness of catchers under low mosquito densities, or greater vigilance at higher densities [15, 26, 28, 30, 55, 56]. The limits of agreement representing the ratios of geometric mean catches per village-month to the HLC were quite wide, although this declined with increasing abundance of mosquitoes. This variability would be expected to reduce if the comparative estimates are aggregated at periods longer than a month and for areas larger than the villages of this study. In the present case, the high variability in the observed ratios presented a challenge to translate *Anopheles* biting rates between the CDC-LT and the indoor HLC. This suggests CDC-LT would not work well as HLC proxies for estimating anopheline man-biting rates, at least on the scale of village-months.

The findings do not imply that the CDC-LT and the HDT are not important. For sampling mosquito populations of an area, the CDC-LT has been found to be very useful [22, 26–28]. This is useful where the absolute numbers of mosquitoes are not required for instance in comparing treatment arms in a trial or monitoring densities over time. The CDCLT is also useful for estimating the SR. Because the SR are usually low, large numbers of mosquitoes are required to estimate them and this can be impractical with HLC. However some caution is needed. Some studies have found that the CDC-LT catches a mosquito sample with a higher SR than the HLC [26, 29]. This may be explained by the tendency for CDC-LT to collect resting [57] and older [58] members of populations. Both factors have been associated with a higher SR as resting mosquitoes have blood fed and older mosquitoes are more likely to have completed the extrinsic incubation period of the parasite. Nevertheless, combining CDC-LT estimated SRs and the HLC biting rates can be a practical way to estimate EIRs.

The HDT has demonstrated good prospects in sampling a range of outdoor biting populations [33, 35]. With the current scarcity of effective traps for monitoring exophagic populations, the HDT may be a candidate for improvement. Generally low HDT catches in this study including those of the local *An. arabiensis* populations known to predominantly bite and rest outdoors may have been because of the operational challenges of using the trap at field scale. The field team of this study reported difficulties in transporting and setting up the traps from location to location and in obtaining and heating large volumes of water in the remote setting where the trial was conducted. They also found it time consuming to remove mosquitoes from the sticky acetate that is a component of the trap.

Different studies of the CDC-LT and HDT summarized in Table 1 reveal important factors influencing the trap performances. Key among these is the species composition and behaviours of local populations. In the present study, both the CDC-LT and HDT performed poorer than the HLC independent of mosquito species despite significant variabilities in the numbers caught (Fig. 4 and Table 2). Similar observations were made in other areas where the CDC-LT under- [59] or out-performed [15, 60] the HLC, independent of the mosquito species. However, in other settings trap performances differed based on species type [20, 30]. It is worth noting that species-specific differences of the CDC-LT performance are not often clear since the exact mechanism of light attraction of mosquitoes is not properly known [61]. Light attraction is thought to be loosely linked to human biting [24]. A high CDC-LT *An. funestus* catch in this study reflects the predominance of anthropophagic and endophilic behaviours of the local populations as has previously been reported in the same area [37, 38]. Other observed sources of variation include location [32], whether the traps are indoors or outdoors [55, 58] and the presence or absence of ITNs [27]. Overgaard et al. [55] observed that the results of CDC-LT efficacy may also vary based on the different statistical analyses employed. This underscores the need to be aware and to reconcile possible methodological inconsistencies while evaluating traps. The choice of host decoy i.e. whether cow or human [35], location [32, 33] and seasonality [31] are among the factors observed to influence the HDT performance.

In summary, considering the rich diversities of the Afrotropical vector populations adapting to their local environments, and the paramount need to obtain accurate and locally relevant entomological metrics, the choice of appropriate monitoring tools follows a use-case basis that should respect local settings. Of great importance is to appreciate the limitations of the individual tools. The HLC remains the most accurate tool for obtaining the epidemiologically-relevant entomologic metrics of the man-biting rates, used as a component of EIR. However, the use of the CDC-LT is extremely useful for population level estimates of SR and for monitoring relative changes in mosquito density through time, for instance, in response to control tools. The CDC-LT if used cautiously with case-by-case appreciation of its limitations could be a suitable entomological surveillance tool for indoor foraging mosquitoes, particularly in light of the safety concerns with HLC. The tendency of the traps to under- or oversample host-seeking anophelines could potentially be resolved by regression methods with reference to the HLC, as long as the limits of agreement are reasonably narrow. The traps are more objective since they are less prone to human sources of error, they are more acceptable within households than catchers visiting at night, and are convenient to deploy on largescale [22, 26]. Improvements on the HDT’s current design are necessary to make the trap more effective [32]. Meanwhile, the CDC-LTs adapted for outdoor surveys [32, 62] and the furvela tent trap (FTT) [32, 63] are some of the possible alternatives for outdoor biting surveys. For both indoor and outdoor surveys where necessary, limited use of the HLC is advocated.

## Conclusion

CDC-LT can be suitable for comparing mosquito populations between study arms or over time if accuracy in the absolute biting rate, compared to HLC, is not required. CDC-LT is useful for estimating sporozoite rates because large numbers of traps can be deployed to collect adequate mosquito samples. The present design of the HDT is not amenable for use in large-scale entomological surveys. Use of HLC remains important for estimating human exposure to mosquitoes as part of estimating the entomological inoculation rate (EIR).

## Supplementary Information

**Additional file 1.** Additional tables.

## Data Availability

The datasets used and or analysed in this study are available from the corresponding author upon reasonable request.
