# Supplementary material for: The Centres for Disease Control light trap (CDC-LT) and the human decoy trap (HDT) compared to the human landing catch (HLC) for measuring Anopheles biting in rural Tanzania
Source: Malar J. 2022 Jun 11;21:181. doi: 10.1186/s12936-022-04192-9 (PMC9188237; doi:10.1186/s12936-022-04192-9)
Supplement: Supplementary file 1 — Additional file 1. Additional tables. [file 12936_2022_4192_MOESM1_ESM.docx]

# Additional files

| Additional file 1: Table S1. Household and study area factors. |
| --- |
| \|  \| \|  \|  \|  \|  \|  \|  \|  \|  \|  \|  \|  \|  \| \| --- \| --- \| --- \| --- \| --- \| --- \| --- \| --- \| --- \| --- \| --- \| --- \| --- \| --- \| \| Baseline factors \| \| **Study 1** \| \| \|  \|  \| **Study 2** \| \| \| \| \| \| \| \|  \| \|  \|  \|  \|  \|  \|  \|  \|  \|  \|  \|  \|  \| \|  \| \| **Indoor surveys** \| \| \| \| \| \| \| \|  \| **Outdoor surveys** \| \| \| \|  \| \|  \|  \|  \|  \|  \|  \|  \|  \|  \|  \|  \|  \| \|  \| \| **CDC-LT** \|  \| **Indoor HLC** \|  \|  \| **CDC-LT** \|  \| **Indoor HLC** \|  \| **Outdoor HLC** \|  \| **HDT** \| \|  \| \|  \|  \|  \|  \|  \|  \|  \|  \|  \|  \|  \|  \| \| Number of households \| \| 214 \|  \| 68 \|  \|  \| 542 \|  \| 39 \|  \| 39 \|  \| 71 \| \|  \| \|  \|  \|  \|  \|  \|  \|  \|  \|  \|  \|  \|  \| \| Household size \| \|  \|  \|  \|  \|  \|  \|  \|  \|  \|  \|  \|  \| \|  \| *≤ 5 members* \| 154 (72%) \|  \| 47 (69%) \|  \|  \| 462 (85%) \|  \| 34 (87%) \|  \| 34 (87%) \|  \| 61 (86%) \| \|  \| *> 5 members* \| 60 (28%) \|  \| 21 (31%) \|  \|  \| 80 (15%) \|  \| 5 (13%) \|  \| 5 (13%) \|  \| 10 (14%) \| \| Total number of households (N) \| \| 214 (100%) \|  \| 68 (100%) \|  \|  \| 542 (100%) \|  \| 39 (100%) \|  \| 39 (100%) \|  \| 71 (100%) \| \|  \| \|  \|  \|  \|  \|  \|  \|  \|  \|  \|  \|  \|  \| \| Seasonality of collections \| \|  \|  \|  \|  \|  \|  \|  \|  \|  \|  \|  \|  \| \|  \| *long rains (Jan-May)* \| 439 (81%) \|  \| 141 (98%) \|  \|  \| 3280 (60%) \|  \| 119 (61%) \|  \| 119 (61%) \|  \| 25 (18%) \| \|  \| *dry season (Jul-Dec)* \| 100 (19%) \|  \| 3 (2%) \|  \|  \| 2194 (40%) \|  \| 76 (39%) \|  \| 76 (39%) \|  \| 111 (82%) \| \| Total collections (N) \| \| 539 (100%) \|  \| 144 (100%) \|  \|  \| 5474 (100%) \|  \| 195 (100%) \|  \| 195 (100%) \|  \| 136 (100%) \| \|  \| \|  \|  \|  \|  \|  \|  \|  \|  \|  \|  \|  \|  \| \| IRS treatment \| \| \|  \|  \|  \|  \|  \|  \|  \|  \|  \|  \|  \| \|  \| *Intervention arm* \| 307 (57%) \|  \| 71 (49%) \|  \|  \| 2544 (46%) \|  \| 97 (50%) \|  \| 97 (50%) \|  \| 64 (47%) \| \|  \| *Control arm* \| 232 (43%) \|  \| 73 (51%) \|  \|  \| 2930 (54%) \|  \| 98 (50%) \|  \| 98 (50%) \|  \| 72 (53%) \| \| Total collections (N) \| \| 539 (100%) \|  \| 144 (100%) \|  \|  \| 5474 (100%) \|  \| 195 (100%) \|  \| 193 (100%) \|  \| 136 (100%) \| \|  \|  \|  \|  \|  \|  \|  \|  \|  \|  \|  \|  \|  \|  \| \| Persons per ITN in households \| \|  \|  \|  \|  \|  \|  \|  \|  \|  \|  \|  \|  \| \|  \| *No ITNs* \| 7 (3%) \|  \| 2 (3%) \|  \|  \| 14 (3%) \|  \| 3 (8%) \|  \| 3 (8%) \|  \| 3 (4.2%) \| \|  \| *1 ITN/≤2 persons* \| 121 (57%) \|  \| 35 (51%) \|  \|  \| 359 (66%) \|  \| 29 (74%) \|  \| 29 (74%) \|  \| 45 (63.4%) \| \|  \| *1 ITN/>2 persons* \| 93 (43%) \|  \| 33 (49%) \|  \|  \| 169 (31%) \|  \| 7 (18%) \|  \| 7 (18%) \|  \| 23 (32.4%) \| \| Total number of households (N) \| \| 214 (100%) \|  \| 68 (100%) \|  \|  \| 542 (100%) \|  \| 39 (100%) \|  \| 39 (100%) \|  \| 71 (100%) \| \|  \| \|  \|  \|  \|  \|  \|  \|  \|  \|  \|  \|  \|  \| \| House screening* \| \|  \|  \|  \|  \|  \|  \|  \|  \|  \|  \|  \|  \| \|  \| *no mosquito proofing* \| 196 (36%) \|  \| 70 (48.6%) \|  \|  \| 2863 (52%) \|  \| 111 (57%) \|  \| 111 (57%) \|  \| 69 (51%) \| \|  \| *mosquito proofing* \| 343 (64%) \|  \| 74 (51.4%) \|  \|  \| 2611 (48%) \|  \| 84 (43%) \|  \| 84 (43%) \|  \| 67 (49%) \| \| Total collections (N) \| \| 539 (100%) \|  \| 144 (100%) \|  \|  \| 5474 (100%) \|  \| 195 (100%) \|  \| 195 (100%) \|  \| 136 (100%) \| \|  \|  \|  \|  \|  \|  \|  \|  \|  \|  \|  \|  \|  \|  \| \| Livestock and pets \| \|  \|  \|  \|  \|  \|  \|  \|  \|  \|  \|  \|  \| \|  \| *No animals* \| 70 (33%) \|  \| 21 (31%) \|  \|  \| 12 (2%) \|  \| 1 (3%) \|  \| 1 (3) \|  \| 2 (3%) \| \|  \| *Poultry, cats and dogs only* \| 109 (51%) \|  \| 34 (50%) \|  \|  \| 246 (45%) \|  \| 15 (38%) \|  \| 15 (38%) \|  \| 33 (38%) \| \|  \| *At least goat, donkey or cow* \| 35 (16%) \|  \| 13 (19%) \|  \|  \| 20 (4%) \|  \| 2 (5%) \|  \| 2 (5%) \|  \| 2 (5%) \| \|  \| *Not recorded* \| 0 (0%) \|  \| 0 (0%) \|  \|  \| 264 (49%) \|  \| 21 (54%) \|  \| 21 (54%) \|  \| 34 (54%) \| \| Total number of households (N) \| \| 214 (100%) \|  \| 68 (100%) \|  \|  \| 542 (100%) \|  \| 39 (100%) \|  \| 39 (100%) \|  \| 71 (100%) \| \|  \| \|  \|  \|  \|  \|  \|  \|  \|  \|  \|  \|  \|  \| \| These factors were added as covariates in the multivariable negative binomial-GLMMs to account for associated variability of mosquito densities and resultant influence upon the efficacy of traps.  *House screening was coded on the basis of window screens and eaves condition as follows: 1. not mosquito proofed = open eaves + no window screening, 2. partially mosquito proofed = closed eaves or screened windows, 3. mosquito proofed = screened windows + closed eaves \| \| \| \| \| \| \| \| \| \| \| \| \| \| |

| Additional file 1: Table S2. Estimated rate ratios and 95% CI of indoor biting mosquito densities for trap type and other factors. |
| --- |
| \|  \|  \|  \|  \|  \|  \|  \|  \|  \|  \|  \|  \|  \| \| --- \| --- \| --- \| --- \| --- \| --- \| --- \| --- \| --- \| --- \| --- \| --- \| --- \| \|  \|  \| ***An. arabiensis*** \| \| \|  \| ***An. funestus*** \| \| \|  \| ***Culex* spp** \| \| \| \| Covariates \|  \|  \|  \|  \|  \|  \|  \|  \|  \|  \|  \|  \| \|  \|  \| Adj.RR (95%CI) \|  \| *p value* \|  \| Adj.RR (95%CI) \|  \| *p value* \|  \| Adj.RR (95%CI) \|  \| *P value* \| \|  \|  \|  \|  \|  \|  \|  \|  \|  \|  \|  \|  \|  \| \| Trap type \| 1 = Human landing catch \| 1* \|  \|  \|  \| 1* \|  \|  \|  \| 1* \|  \|  \| \|  \| 2 = CDC-LT \| 0.35 (0.27-0.46) \|  \| *< 0.001* \|  \| 0.63 (0.51-0.79) \|  \| *< 0.001* \|  \| 0.82 (0.67-1.01) \|  \| *0.061* \| \|  \|  \|  \|  \|  \|  \|  \|  \|  \|  \|  \|  \|  \| \|  \|  \|  \|  \|  \|  \|  \|  \|  \|  \|  \|  \|  \| \|  \|  \|  \|  \|  \|  \|  \|  \|  \|  \|  \|  \|  \| \| Household size \|  \|  \|  \|  \|  \|  \|  \|  \|  \|  \|  \|  \| \|  \| 1 = ≤ 5 members \| 1* \|  \| *0.220* \|  \| 1* \|  \| *0.439* \|  \| 1* \|  \| *0.481* \| \|  \| 2 = > 5 members \| 0.86 (0.68-1.09) \|  \|  \|  \| 0.93 (0.78-1.12) \|  \|  \|  \| 1.08 (0.88-1.33) \|  \|  \| \|  \|  \|  \|  \|  \|  \|  \|  \|  \|  \|  \|  \|  \| \| Study (1&2) category \|  \|  \|  \|  \|  \|  \|  \|  \|  \|  \|  \|  \| \|  \| 1 = Study 1 (2017) \| 1* \|  \| *0.065* \|  \| 1* \|  \| *< 0.001* \|  \| 1* \|  \| *0.082* \| \|  \| 2 = Study 2 (2019) \| 0.63 (0.38-1.02) \|  \|  \|  \| 7.85 (5.34-11.52) \|  \|  \|  \| 1.33 (0.96-1.82) \|  \|  \| \|  \|  \|  \|  \|  \|  \|  \|  \|  \|  \|  \|  \|  \| \| Seasons \|  \|  \|  \|  \|  \|  \|  \|  \|  \|  \|  \|  \| \|  \| 1 = long rains \| 1* \|  \| *< 0.001* \|  \| 1* \|  \| *0.002* \|  \| 1* \|  \| *0.006* \| \|  \| 2 = dry season \| 0.07 (0.04-0.10) \|  \|  \|  \| 0.64 (0.49-0.84) \|  \|  \|  \| 0.82 (0.72-0.94) \|  \|  \| \|  \|  \|  \|  \|  \|  \|  \|  \|  \|  \|  \|  \|  \| \| Indoor residual spraying \|  \|  \|  \|  \|  \|  \|  \|  \|  \|  \|  \|  \| \|  \| 0 = positive control \| 1* \|  \| *< 0.001* \|  \| 1* \|  \| *< 0.001* \|  \| 1* \|  \| *< 0.001* \| \|  \| 1 = IRS product 1 \| 1.13 (0.74-1.71) \|  \|  \|  \| 1.80 (1.27-2.57) \|  \|  \|  \| 0.66 (0.48-0.92) \|  \|  \| \|  \| 2 = IRS product 2 \| 0.52 (0.43-0.62) \|  \|  \|  \| 2.67 (2.33-3.07) \|  \|  \|  \| 1.66 (1.41-1.96) \|  \|  \| \|  \|  \|  \|  \|  \|  \|  \|  \|  \|  \|  \|  \|  \| \| Insecticide treated nets \|  \|  \|  \|  \|  \|  \|  \|  \|  \|  \|  \|  \| \|  \| 1 = 1 ITN/≤2 persons \| 1* \|  \| *0.940* \|  \| 1* \|  \| *0.012* \|  \| 1* \|  \| *0.630* \| \|  \| 2 = 1 ITN/>2 persons & no ITNs \| 0.99 (0.82-1.20) \|  \|  \|  \| 0.84 (0.73-0.96) \|  \|  \|  \| 1.96 (0.82-1.13) \|  \|  \| \|  \|  \|  \|  \|  \|  \|  \|  \|  \|  \|  \|  \|  \| \| House screening \|  \|  \|  \|  \|  \|  \|  \|  \|  \|  \|  \|  \| \|  \| 1 = not mosquito proofed \| 1* \|  \| *0.243* \|  \| 1* \|  \| *0.604* \|  \| 1* \|  \| *0.702* \| \|  \| 2 = mosquito proofed \| 0.90 (0.76-1.07) \|  \|  \|  \| 0.97 (0.85-1.10) \|  \|  \|  \| 0.97 (0.84-1.13) \|  \|  \| \|  \|  \|  \|  \|  \|  \|  \|  \|  \|  \|  \|  \|  \| \| Livestock and pets \|  \|  \|  \|  \|  \|  \|  \|  \|  \|  \|  \|  \| \|  \| 1 = none \| 1* \|  \| *0.472* \|  \| 1* \|  \| *< 0.001* \|  \| 1* \|  \| *0.126* \| \|  \| 2 = poultry, dogs, cats only \| 1.09 (0.78-1.54) \|  \|  \|  \| 1.06 (0.80-1.40) \|  \|  \|  \| 0.86 (0.64-1.16) \|  \|  \| \|  \| 3 = At least goat, cow or donkey \| 0.82 (0.51-1.31) \|  \|  \|  \| 0.59 (0.40-0.87) \|  \|  \|  \| 1.92 (0.62-1.36) \|  \|  \| \|  \| 4 = Not recorded \| 1.13 (0.78-1.63) \|  \|  \|  \| 0.86 (0.64-1.15) \|  \|  \|  \| 1.05 (0.76-1.46) \|  \|  \| \| 1* refers to the reference method or category  The single p values for the categorical variables were estimated by the likelihood ratio test  CI = Confidence interval  Adj.RR = Adjusted rate ratios estimated from multivariable mixed effects regression models \| \| \| \| \| \| \| \| \| \| \| \| \| |

| Additional file 1: Table S3. Estimated rate ratios and 95% CI of outdoor biting mosquito densities for trap type and other factors. |
| --- |
| \|  \|  \|  \|  \|  \|  \|  \|  \|  \|  \|  \|  \|  \| \| --- \| --- \| --- \| --- \| --- \| --- \| --- \| --- \| --- \| --- \| --- \| --- \| --- \| \|  \|  \| ***An. arabiensis*** \| \| \|  \| ***An. funestus*** \| \| \|  \| ***Culex* spp** \| \| \| \| Covariates \|  \|  \|  \|  \|  \|  \|  \|  \|  \|  \|  \|  \| \|  \|  \| Adj.RR (95%CI) \|  \| *p value* \|  \| Adj.RR (95%CI) \|  \| *p value* \|  \| Adj.RR (95%CI) \|  \| *P value* \| \|  \|  \|  \|  \|  \|  \|  \|  \|  \|  \|  \|  \|  \| \| Trap type \| 1 = Human landing catch \| 1* \|  \|  \|  \| 1* \|  \|  \|  \| 1* \|  \|  \| \|  \| 3 = Host decoy trap \| 0.04 (0.01-0.14) \|  \| *< 0.001* \|  \| 0.10 (0.07-0.15) \|  \| *< 0.001* \|  \| 0.20 (0.14-0.29) \|  \| *< 0.001* \| \|  \|  \|  \|  \|  \|  \|  \|  \|  \|  \|  \|  \|  \| \| Household size \|  \|  \|  \|  \|  \|  \|  \|  \|  \|  \|  \|  \| \|  \| 1 = ≤ 5 members \| 1* \|  \| *0.454* \|  \| 1* \|  \| *0.068* \|  \| 1* \|  \| *0.348* \| \|  \| 2 = > 5 members \| 1.52 (0.52-4.45) \|  \|  \|  \| 1.68 (0.96-2.93) \|  \|  \|  \| 1.27 (0.77-2.09) \|  \|  \| \|  \|  \|  \|  \|  \|  \|  \|  \|  \|  \|  \|  \|  \| \| Season \|  \|  \|  \|  \|  \|  \|  \|  \|  \|  \|  \|  \| \|  \| 1 = long rains \| 1* \|  \| *< 0.001* \|  \| 1* \|  \| *< 0.001* \|  \| 1* \|  \| *0.007* \| \|  \| 2 = dry season \| 0.05 (0.02-0.12) \|  \|  \|  \| 0.57 (0.46-0.71) \|  \|  \|  \| 0.73 (0.59-0.91) \|  \|  \| \|  \|  \|  \|  \|  \|  \|  \|  \|  \|  \|  \|  \|  \| \| Indoor residual spraying \|  \|  \|  \|  \|  \|  \|  \|  \|  \|  \|  \|  \| \|  \| 0 = positive control \| 1* \|  \| *0.032* \|  \| 1* \|  \| *< 0.001* \|  \| 1* \|  \| *0.172* \| \|  \| 2 = IRS product 2 \| 0.47 (0.23-0.95) \|  \|  \|  \| 2.50 (1.68-3.70) \|  \|  \|  \| 1.27 (0.90-1.80) \|  \|  \| \|  \|  \|  \|  \|  \|  \|  \|  \|  \|  \|  \|  \|  \| \| Insecticide treated nets \|  \|  \|  \|  \|  \|  \|  \|  \|  \|  \|  \|  \| \|  \| 1 = 1 ITN/≤2 persons \| 1* \|  \| *0.668* \|  \| 1* \|  \| *0.325* \|  \| 1* \|  \| *0.861* \| \|  \| 2 = 1 ITN/>2 persons & no ITNs \| 1.20 (0.52-2.78) \|  \|  \|  \| 0.80 (0.51-1.26) \|  \|  \|  \| 0.97 (0.65-1.43) \|  \|  \| \|  \|  \|  \|  \|  \|  \|  \|  \|  \|  \|  \|  \|  \| \| Livestock and pets \|  \|  \|  \|  \|  \|  \|  \|  \|  \|  \|  \|  \| \|  \| 1 = none \| 1* \|  \| *0.439* \|  \| 1* \|  \| *0.611* \|  \| 1* \|  \| *0.399* \| \|  \| 2 = poultry, dogs, cats only \| 3.25 (0.40-26.52) \|  \|  \|  \| 1.49 (0.46-4.81) \|  \|  \|  \| 0.67 (0.25-1.81) \|  \|  \| \|  \| 3 = At least goat, cow or donkey \| 7.61 (0.64-90.05) \|  \|  \|  \| 2.01 (0.47-8.52) \|  \|  \|  \| 0.33 (0.09-1.25) \|  \|  \| \|  \| 4 = Not recorded \| 2.97 (0.39-22.59) \|  \|  \|  \| 1.22 (0.38-3.88) \|  \|  \|  \| 0.63 (0.24-1.70) \|  \|  \| \| 1* refers to the reference method or category  The single p values for the categorical variables were estimated by the likelihood ratio test  CI = Confidence interval  Adj.RR = Adjusted rate ratios estimated from multivariable mixed effects regression models \| \| \| \| \| \| \| \| \| \| \| \| \| |

Additional file 1: Table S4. Relative species abundance per trap per night

|  |  | | | | | | | | | | | | | | |
| --- | --- | --- | --- | --- | --- | --- | --- | --- | --- | --- | --- | --- | --- | --- | --- |
|  | **CDC-LT** | | |  | **Indoor HLC** | | |  | **HDT** | | |  | **Outdoor HLC** | | |
|  | Adj.RR  (95% CI) |  | p value |  | Adj.RR  (95% CI) |  | p value |  | Adj.RR  (95% CI) |  | p value |  | Adj.RR  (95% CI) |  | p value |
| *An. arabiensis* | 1* |  |  |  | 1* |  |  |  | 1* |  |  |  | 1* |  |  |
|  |  |  |  |  |  |  |  |  |  |  |  |  |  |  |  |
| *An. funestus* | 9.05  (7.95-10.14) |  | < 0.001 |  | 8.93  (7.84-10.02) |  | < 0.001 |  | 6.95  (5.51-8.38) |  | < 0.001 |  | 2.44  (3.79-1.09) |  | < 0.001 |
| The relative species abundance per trap per night (Adj.RR and 95% CIs) was estimated from multivariable negative binomial-GLMMs, regressing mosquito counts on the species type.  1* refers to the reference species | | | | | | | | | | | | | | | |
